# Uncorrelated Age-Related Changes in Visuo-Spatial Working Memory Binding and Thermoregulation

**DOI:** 10.3390/clockssleep7020017

**Published:** 2025-03-22

**Authors:** Marine Dourte, Gregory Hammad, Christina Schmidt, Philippe Peigneux

**Affiliations:** 1 Sleep and Chronobiology Laboratory, GIGA-CRC Human Imaging Unit, University of Liège, 4000 Liège, Belgium; marine.dourte@uliege.be (M.D.); gregory.hammad@uliege.be (G.H.); philippe.peigneux@ulb.be (P.P.); 2 Psychology and Neuroscience of Cognition Research Unit (PsyNCog), Faculty of Psychology and Educational Sciences, University of Liège, 4000 Liège, Belgium; 3 UR2NF, Neuropsychology and Functional Neuroimaging Research Unit, Affiliated at Center for Research in Cognition and Neurosciences and ULB Neurosciences Institute, University Libre de Bruxelles (ULB), 1050 Brussels, Belgium

**Keywords:** circadian rhythms, visuo-spatial working memory, ageing, thermoregulation, distal–proximal gradient (DPG), cognitive performance

## Abstract

Ageing is associated with alterations in circadian rhythms and thermoregulation, contributing to a fragmentation of the sleep–wake cycle and possibly age-related changes in cognitive performance. In this study, we investigated the relationship between visuo-spatial working memory (vsWM) performance and thermoregulation in young (18–34 years) and old (64–84 years) healthy human adults. Variations in the distal–proximal skin temperature gradient (DPG) were continuously monitored over the 24 h cycle in a field setting. vsWM was assessed during morning (09:00) and evening sessions (17:00) using an object–location binding task. As expected, a reduced circadian DPG amplitude was observed in old as compared to young participants. Likewise, old participants produced more errors than the young ones in object identification and location, suggesting reduced vsWM ability. Notwithstanding this, no significant association was found between circadian DPG modulation and vsWM performance, nor between testing time-of-day and cognitive performance. Further research is needed to explore environmental factors and the timing of peak circadian rhythms to better understand the interplay between circadian biology and cognitive ageing.

## 1. Introduction

Circadian rhythms evolve across the human lifespan [1,2,3]. Amongst other changes, ageing has been associated with lower heat production and conservation, impaired heat loss, and reduced thermal sensitivity [4,5,6]. These alterations have been suggested to contribute to the classically observed phase advance and reduced circadian amplitude of the core body temperature (CBT), as well as to overall increased daytime distal skin temperature [5]. The distal–proximal gradient (DPG, computed as the subtraction of the mean proximal skin temperature from the mean distal skin temperature), which indirectly accounts for heat redistribution from the core to the periphery and daily dynamics in thermoregulation, is viewed as a sensitive marker for circadian phase and amplitude in humans [7,8,9,10]. Indeed, the circadian modulation of proximal skin temperature was positively linked to that of CBT, while the circadian rhythms of distal skin temperature were inversely correlated to the CBT circadian modulation [11,12]. DPG therefore exhibits circadian modulation throughout the sleep–wake cycle. While an increase in skin temperature is associated with faster sleep onset and heightened sleepiness [11,12,13], some studies show that its circadian modulation is partially unaffected by the homeostatic buildup of sleep pressure [12,14]. Instead, it serves as a signal to the brain, indicating readiness for sleep [11,15]. This makes DPG a convenient, non-invasive way to explore circadian organisation in individuals [5,7,8].

Ageing is characterised by changes in physiological and behavioural parameters that can in turn exert a detrimental effect on cognitive performance [16,17]. In the memory domain, visuo-spatial working memory (vsWM, which allows temporary maintenance of visual information while the direct input is lacking) was shown to be affected by age [18,19,20,21,22]. Furthermore, time-of-day, i.e., a circadian effect, was found to modulate these age-related changes [23]. It is assumed that the identity and the location of a target stimulus are separately stored for short amounts of time in vsWM and that the final visual representation is achieved by the process of binding these two different elements [24,25]. While some studies have found the binding process to be unaffected by age in healthy individuals [26,27,28], others have reported an age-related decline [18,19,20,29]. Binding was even proposed as one of the first processes to decline in the early stages of pathological ageing such as in Alzheimer’s disease [27].

Associations have been reported between global working memory performance and daily CBT oscillations [30,31,32,33,34,35], as well as the homeostatic buildup and dissipation of sleep pressure [30]. Daily modulation of performance was found to parallel the CBT curve and inversely mirror DPG [30,31,36,37,38]. Indeed, performance on cognitive tasks is positively correlated with body temperature, being highest during the biological day when CBT is at its peak and DPG is at its trough. An increase of just ~0.15 °C has been associated with significant improvements in cognitive performance and alertness, indicating that even small changes in temperature may be associated with cognitive benefits [6,39]. Furthermore, tasks with a high cognitive load, such as those requiring sustained attention or working memory, seem to be more affected by body temperature changes than simpler reaction-time tasks [39].

In this study, we investigated the association between visuo-spatial binding performance in working memory, circadian DPG modulation, and time-of-day in groups of healthy old and young individuals. We hypothesised a reduced amplitude in the 24 h modulation of DPG in healthy old participants, concomitantly with a poorer performance in the visuo-spatial binding task, as compared to young participants. We further assumed that vsWM performance would be linked to DPG measures during testing times, with a lower DPG being linked to better performance. Furthermore, we speculated that differences in vsWM performance between morning and evening assessments would correlate with DPG differences.

## 2. Results

### 2.1. vsWM Task

#### 2.1.1. Identification Performance

**Figure 1:**
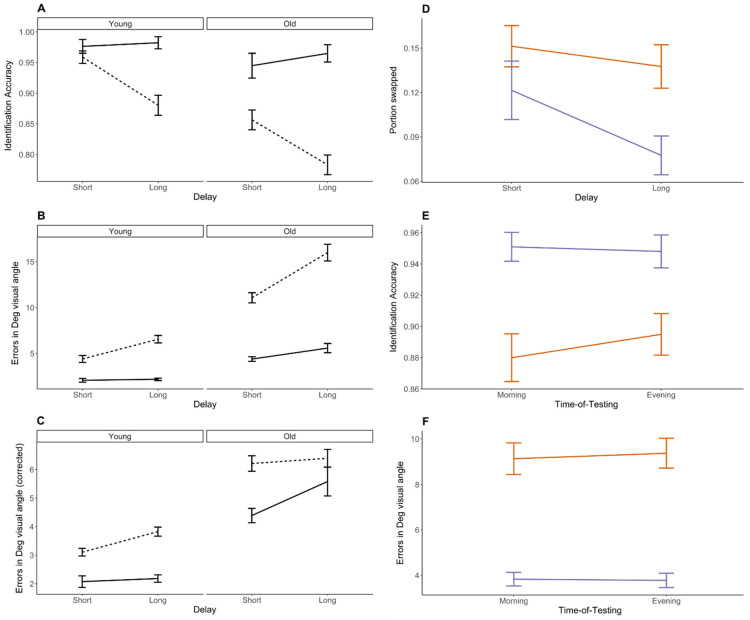
Performance in the visuo-spatial object-binding task in old and young participants. (**A**,**E**) Identification accuracy; (**B**,**F**) location errors (visual angle degree); (**C**) NIC, i.e., location error controlled for object–location swapping; (**D**) proportion of swap errors. Left panel: number of items is depicted in full lines for 1 item and in dashed lines for 3 items. Right panel: old participants are depicted in orange and young participants in purple. Individual data points are depicted for the older group in Figure S1 of the Supplementary Materials.

The proportion of correctly identified targets in each group is depicted Figure 1A. The analysis highlighted the main effects of maintenance delay (*F* = 26.55, *p* < 0.001, η^2^ = 0.019) and number of items (*F* = 156.63, *p* < 0.001, η^2^ = 0.198), with identification accuracy being poorer with a longer maintenance delay and a higher number of items. The delay*number of items interaction effect was significant (*F* = 34.58, *p* < 0.001, η^2^ = 0.039); post hoc tests showed that long delays and more items led to poorest performance (*t_279_* = −10.84, *p* < 0.001). Additionally, there was a main group effect (*F* = 64.28, *p* < 0.001, η^2^ = 0.075) with overall poorer accuracy in old than young participants. Finally, the group*number of items interaction was significant (*F* = 36.58, *p* < 0.001, η^2^ = 0.028); post hoc tests indicate that keeping more items in vsWM impacts old more than young participants (*t* = −5.68, *p* < 0.001). Main time-of-testing (*F* = 0.033, *p* = 0.57, η^2^ < 0.01) and group*time-of-testing (*F* = 3.32, *p* = 0.07, η^2^ < 0.01) effects were non-significant. Identification performance by time-of-testing and group is depicted in Figure 1E.

#### 2.1.2. Absolute Location Error

Absolute location errors per group are depicted in Figure 1B. The main effects of maintenance delay (*F* = 66.53, *p* < 0.001, η^2^ = 0.040) and number of items (*F* = 339.81, *p* < 0.001, η^2^=0.321), and their interaction (*F* = 43.74, *p* < 0.001, η^2^ = 0.019), were significant. Post hoc tests showed poorer performance at longer delays (*t* = 8.25, *p* < 0.001) and with a higher number of items displayed (*t* = −18.40, *p* < 0.001), as well as with a combination of the two (*t* = 17.20, *p* < 0.001). Old participants produced more location errors than young participants (main group effect, *F* = 295.90, *p* < 0.001, η^2^ = 0.250). Significant interactions were observed between group and delay (*F* = 12.887, *p* < 0.001, η^2^ = 0.008), as well as group and number of items (*F* = 98.444, *p* < 0.001, η^2^ = 0.057). Old participants had more errors than young participants at both increased delay (*t* = 12.97, *p* < 0.001) and number of items (*t* = 12.38, *p* < 0.001). There were no main time-of-testing (*F* = 0.089, *p* = 0.77, η^2^ < 0.01) nor group*time-of-testing interaction (*F* = 0.036, *p* = 0.85, η^2^ < 0.01) effects. Absolute location error by time-of-testing and group is depicted Figure 1F.

#### 2.1.3. Location Error Controlling for Swap Errors

A main group effect was observed for the number of swap errors (*F* = 11.97, *p* < 0.001, η^2^ = 0.054), but the interaction with maintenance delay (group*delay, *F* = 0.64, *p* = 0.43, η^2^ = 0.006) or time-of-testing (group*time-of-testing, *F* = 2.72, *p* = 0.10, η^2^ = 0.009) was non-significant. Old individuals made more swap errors than young participants. Time-of-testing (*F* = 0.001, *p* = 0.97, η^2^ = 0.001) and delay (*F* = 2.96, *p* = 0.09, η^2^ = 0.020) did not significantly affect swap errors. Location errors controlled for object–location swaps (NIC) are depicted in Figure 1C, and the number of swap errors per group and maintenance delay are shown in Figure 1D.

When location errors were controlled for swap errors, the analysis disclosed the main effects of delay (*F* = 7.68, *p* = 0.006, η^2^ = 0.014), number of items (*F* = 91.29, *p* < 0.001, η^2^ = 0.079) and group (*F* = 346.86, *p* < 0.001, η^2^ = 0.363). Whereas old participants exhibited an overall higher error rate than the young participants, performance was not modulated by longer maintenance delay (group*delay interaction, *F* = 0.20, *p* = 0.66, η^2^ < 0.01) nor by a higher number of items (group*number of item interaction, *F* = 0.07, *p* = 0.79, η^2^ < 0.01). Time-of-testing (*F* = 0.10, *p* = 0.75, η^2^ < 0.01) and the interaction with the group factor (*F* = 1.81, *p* = 0.18, η^2^ < 0.01) were non-significant.

### 2.2. The 24 H Time Course of DPG Levels

**Figure 2:**
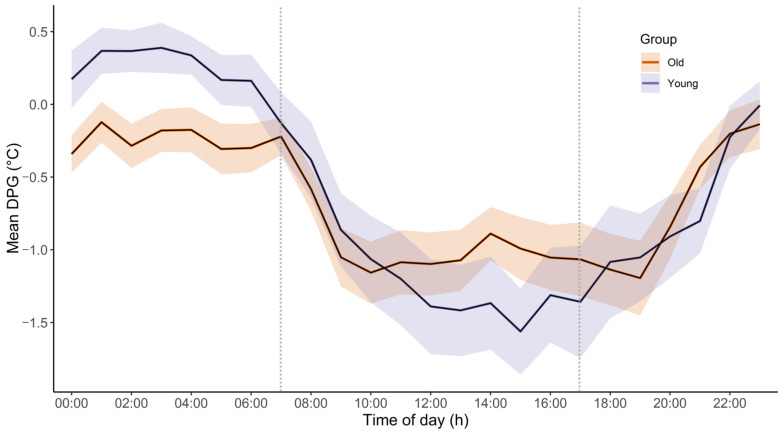
Time course of distal–proximal gradient (DPG) per group (old: orange; young: purple) across the 24 h cycle. SEM are displayed in shaded colour. Testing times are indicated by grey dotted vertical lines.

The 24-h time course of DPG by group is depicted in Figure 2. Analyses disclosed a main time effect (*F* = 18.00, *p* < 0.001, η^2^ = 0.02), indicating DPG modulation over the 24 h cycle. The main group effect was not significant (*F* = 0.14, *p* = 0.71, η^2^ < 0.01), but there was a group * time interaction effect (*F* = 2.07, *p* < 0.001, η^2^ = 0.50), indicating differential DPG modulation throughout the 24 h cycle between the two age groups. Both the group*cosine function interaction (*t* = 4.719, *p* < 0.001, β = 0.339) and the group*sine function (*t* = 2.623, *p* = 0.009, β = 0.297) were significant, substantiating a difference in DPG amplitude and phase over the course of the biological day, with old participants exhibiting a lower amplitude variation (1.03 °C) than their young counterparts (1.73 °C), as well as a 2° (=8 min) phase advance (old participants: 37°; young participants: 39°).

### 2.3. Link Between DPG Levels and Working Memory Performance

Models investigating the influence of DPG measures during testing times on vsWM performance showed a significant group effect for all vsWM parameters: identification performance (*t* = 2.439; *p* = 0.018, η^2^ = 0.003), absolute location error (*t* = −7.912; *p* < 0.001, η^2^ = 0.130), number of swap errors (*t* = −2.061, *p* = 0.044, η^2^ = 0.067), and NIC (*t* = −6.282, *p* < 0.001, η^2^=0.101). However, when looking at the link regardless of testing times, we found no significant associations between DPG and identification performance (*t* = 0.984; *p* = 0.33, η^2^ < 0.01), absolute location error (*t* = −0.738, *p* = 0.46, η^2^ < 0.01), number of swap errors (*t* = −1.504, *p* = 0.14, η^2^ = 0.002), and NIC (*t* = −0.738, *p* = 0.46, η^2^ < 0.01). Moreover, no interaction was found between DPG and group for identification performance (*t* = −0.59, *p* = 0.55, η^2^ < 0.01), absolute location error (*t* = −0.48, *p* = 0.64, η^2^ < 0.01), number of swap errors (*t* = −0.46, *p* = 0.65, η^2^ < 0.01), and NIC (*t* = 1.03, *p* = 0.31, η^2^ < 0.01).

In the second step, Kendall’s tau correlations were computed for each score between the morning and evening difference in vsWM performance and the morning and evening difference between DPG measures. No significant correlation emerged between morning and evening differences in DPG and identification performance (*τ* = −0.05, *z* = −0.09, *p* = 0.39), absolute location error (*τ* = 0.06, *z* = 1.15, *p* = 0.25), proportion of swap errors (*τ* = 0.02, *z* = 0.24, *p* = 0.81), and NIC performance (*τ* = 0.09, *z* = 1.65, *p* = 0.10). Of note, when looking at evening and morning evaluations separately, we found that DPG during evening assessments was significantly correlated with absolute location error (*τ* = 0.129, *z* = 2.26, *p* = 0.024) and NIC (*τ* = 0.113, *z* = 1.98, *p* = 0.048). There was also a trend towards a correlation between DPG and absolute location error during the morning assessment (*τ* = −0.11, *z* = −1.87, *p* = 0.06). All other vsWM measures were not significantly correlated with DPG, whatever the time of assessment (all *p_s_* > 10).

## 3. Discussion

Our study aimed to investigate whether an age-related decline in visuo-spatial working memory (vsWM) and binding performance is linked to DPG assessed over a 24 h cycle. To do so, we continuously monitored proximal and distal body skin temperature using ambulatory thermal sensors and assessed vsWM performance using an object–location binding task at two different time points of the day (morning vs. evening).

First, we observed circadian modulation of the DPG over the 24 h cycle, in line with the previous literature [40,41,42,43]. We also found age-related differences in the amplitude of the DPG modulation, with old participants exhibiting a lower amplitude than their young counterparts. Although we focused on an indirect index of heat redistribution and not CBT itself, this reduction in amplitude aligns with existing evidence for age-associated alterations in circadian rhythm parameters, such as a phase advance and a reduced amplitude [1,2,40,43,44]. Additionally, our results indicate a satisfying reliability of temperature distal–proximal recordings obtained in home-based ecological conditions to assess the circadian modulation of skin temperature. Indeed, we observed a clear circadian rhythmicity even though participants wore the device at home, with no constraints regarding heating or thermal exposure like in well-controlled laboratory conditions, confirming that skin temperature displays a strong endogenous component, despite the existence of multiple external influences [12,45].

Second, we found age-related differences in performance in the vsWM object–location binding task. Old participants exhibited poorer identification accuracy and an increased rate of absolute location errors. These findings are in line with prior research highlighting an age-related decline in vsWM [18,19,20,29] (but see [26,27,28] for null results). We also found that the impact of factors such as a maintenance delay and number of items to process is more pronounced in old than young individuals, as previously reported [29]. Ageing is known to be associated with increasing inter-individual differences [46,47], raising the question of whether some of our old participants would exhibit more profound deficits either in terms thermoregulatory and/or working memory, with potential outliers skewing the data distribution and the means. To address this concern, we display the data points of old participants for all cognitive factors in Supplementary Material Figure S1. An inspection of the Figure S1 data shows that no subset of old individuals skewed the mean or were potential outliers, which might be explained by the fact that our old participants were carefully recruited and exhibited good parameters for their age (see Table 1), which may to some extent qualify them as successful ageing persons [46,47] and reduce the inherent age-related variability. Therefore, these results emphasise the vulnerability of vsWM processes to age-related changes and task demands.

Despite the presence of age-related differences both at the cognitive and thermoregulatory levels, we did not observe significant associations between DPG modulation and cognitive performance, suggesting that, at least within the constraints of our study, daily modulation of DPG does not translate into variations in cognitive (vsWM) performance. Moreover, no performance difference between young and old participants was found between evening and morning evaluations. These results do not support the previous literature showing that young adults tend to show better cognitive performance in the evening while old adults typically perform better in the morning due to their respective chronotype and higher alertness levels during those hours [35,50,51]. However, our old participants displayed a moderate morning chronotype, whereas our young group had a neutral chronotype, which might have diluted such effects, potentially shifting performance evenly across the circadian cycle. Additionally, and though it was not within the scope of this paper, it should be noted that sleep pressure affects cognitive performance differently in young and old adults. The latter exhibit a relative resilience to the effects of sleep buildup [52], whereas young adults proportionally exhibit greater cognitive impairments with higher sleep pressure [53,54]. Hence, although it remains speculative, old adults may compensate for performance during the evening, which will eventually not reduce much as compared to their morning evaluation, and young participants may suffer more from a higher sleep buildup in the evening, eventually counteracting their chronotype-driven preference for evening evaluations. It must also be mentioned that while circadian timing influences cognition, especially working memory and executive control, aligning testing times with individual sleep–wake schedules helps reduce chronotype-induced performance enhancement [55]. Following this line of reasoning, and knowing that even a small 0.15 °C increase can be associated with improved cognitive performance [39], it might be that the absence of differences in the present study can be explained by the morning assessment: whereas young people would “suffer” from the testing moment not being aligned with their chronotype, performance would be compensated for by the apparent decline of DPG at this moment, favouring better performance. However, this could not be demonstrated with our current results.

A limitation of the current protocol is that the so-called morning and evening testing times were chosen to accommodate for acceptable hours to visit participants at home, leading to fixed hours (9 a.m. and 5 p.m.) for the morning and evening testing schedules. Consequently, vsWM tests were performed according to time-of-day and not according to circadian phase. Additionally, this study relied solely on the distal–proximal skin temperature gradient, taking proximal temperature at the subclavicular level, which cannot be equated to core body temperature [42,56]. Still, proximal skin areas mainly contain capillaries with nutritive functions, with the circadian time course of blood flow through these capillaries following the time course of core body temperature [12], while distal skin temperature, mainly containing arterio-venous anastomoses, shows an inverse pattern [57]. Although DPG can be used as an indirect index of heart redistribution from the core [7,8], our results should be regarded with circumspection as they rely on proximal skin to distal skin temperature gradients. Moreover, we could not monitor the environmental temperature exposure of our participants during the 5-day recording. It may be speculated that old participants would tend to stay home more than young participants or seek warmer environments [58]. Future ambulatory studies should investigate potential differences in thermal comfort behaviour in relation to DPG recordings, as such variables could impact thermoregulation processes. Indeed, a lower contrast in activity in daily life and less time spent outside, and thus a lower contrast of environmental temperature, may lead to lower thermoregulation differences and partly explain differences in the modulation of DPG amplitude between young and old people in our study [59]. However, given the correlative nature of this study, we cannot generalise our results or draw any strong conclusions. Future research may benefit from a more comprehensive examination of these factors to elucidate the nuanced interplay between circadian rhythms, thermoregulation, and vsWM in ageing.

## 4. Materials and Methods

### 4.1. Sample

Eighteen young (18–34 years) and twenty-three old (64–84 years) participants gave their written informed consent to participate in this study, which was approved by the Psychology Faculty Advisory Ethics Committee of the Universite Libre de Bruxelles (agreement number #155/2020). Exclusion criteria were the presence or history of circadian, sleep, or related disorders, insufficient uncorrected vision, extreme chronotype (MEQ < 31 or >69 [49]), and poor self-reported quality of sleep in the past month (PSQI > 7 [48]). Too short (<6 h) or excessive (>11 h for young, >9 h for old participants) nocturnal sleep duration was also an exclusion criterion [60]. Young female participants were only tested during their follicular phase and old women had to be out of the acute stage of menopause to account for the impact that hormonal changes can have on both cognition and temperature regulation [38,61,62]. Demographic information and between-group differences are depicted Table 1.

### 4.2. Object–Location Binding Task

**Figure 3:**
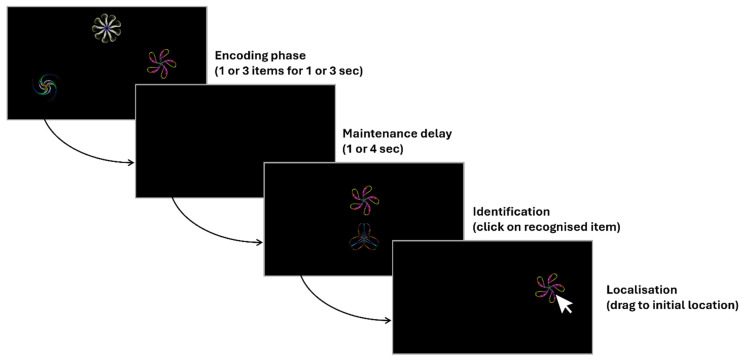
Sample procedure for a 3-item trial, adapted from Pertzov and colleagues [29].

The object–location binding task, created by Pertzov and colleagues [29,63], aims to investigate the binding process linking the identification and spatial components of presented items in visual working memory. As illustrated Figure 3, one or three abstract geometric stimuli (fractals) are presented at specific locations over the screen for one or three seconds, respectively. Then, after a short (1 s) or a long (4 s) delay, one of the presented fractals is shown again in the centre of the screen, together with a new, never presented fractal. Participants are instructed (a) to identify the previously presented fractal by selecting it with the mouse pointer and then (b) to determine its original location by dragging it on its estimated position. When the choice is confirmed by pressing the spacebar, the next trial is initiated [29]. After having completed a 10-trial practice block to ensure correct understanding of the procedure, participants perform 100 trials in a row. To differentiate object identification from spatial location, 3 measures of performance are computed: (1) object identification, i.e., the number of correctly identified items divided by the total number of trials; (2) absolute location errors, i.e., the pixel distance between the reported location of the correctly identified object and its true, original location; and (3) nearest item control (NIC), i.e., a measure of precision in location that considers the possibility of “swap errors”, where individuals correctly identify the fractal but mistakenly report it as being at the location of another item in the memory array. NIC is thus calculated as the distance between the chosen location and the location of the nearest fractal in the original memory array, regardless of whether it was the target item. NIC helps to assess how precisely people remember locations without being concerned about the identity of the items and can help understand location performance irrespective of item identity [29].

### 4.3. Thermometry

Small, flat thermal device probes (iButtons, Maxim/Dallas Semiconductor Corp., Sunnyvale, CA, USA) were applied both to the subclavicular area (proximal skin temperature measurement) and to the wrist of the non-dominant hand (distal skin temperature measurement) using air-permeable plasters (Mefix^®^). A recording was obtained at a 0.0625 °C resolution, averaged over each 300 s epoch. Individual raw recordings were visually inspected, and segments where captors were removed or malfunctioning were excluded from the analysis. The DPG value was obtained by subtracting proximal from distal skin temperature at each epoch, then averaged over 1 h bins, resulting in 24 DPG points per day, reflecting the heat redistribution from proximal skin, an indirect index of CBT (not obtained in this study), to the periphery. These 24 points were then averaged across the 5 recording days to create a 24 h profile per participant.

### 4.4. Experimental Procedure

**Figure 4:**
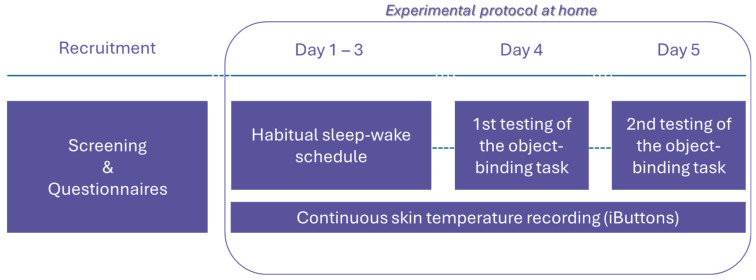
Summary of the study protocol taking place at the participants’ home. Testing times for the object-binding task were either 9 a.m. or 5 p.m. on two different days, counterbalanced.

The experiment took place at the participants’ homes. They were instructed to wear the proximal and distal iButtons for 5 consecutive days (except for periods of extended water immersion, e.g., baths, or high-physical-impact activities). Participants were given precise instructions on how to replace the probes in case of removal. They were also instructed to stick to a regular sleep–wake schedule with no major variations (+/−1 h from pre-selected schedule) and avoid naps. In addition, they were instructed not to consume alcohol and energy drinks/food during the assessment. To facilitate data cleaning and monitoring, they were also asked to fill in a sleep calendar with their approximate wake-up and bedtimes, as well as times and reasons for removing the sensors. On the last 2 days of the protocol, participants were administered the computerised object–location binding task [63]. Testing was conducted at two different times of the day (9 a.m. and 5 p.m.), the administration order being counterbalanced between participants. A schematic representation of the study protocol is depicted Figure 4.

### 4.5. Statistical Analysis

Analyses were conducted using the statistical software R-Studio (R Core Team, 2020), version 4.2.2. Regarding the object–location binding task, identification accuracy and absolute location errors were first analysed separately with the same statistical model, using an Aligned Rank Transform (ART) Anova (ART package, version 0.11.1 [64]), to correct for the non-normal distribution of the data (all *p_s_* < 0.001) and heteroscedasticity (all *p_s_* < 0.001). Both models aimed to investigate the effects of the within-subject factors maintenance delay (2-level factor, short vs. long), number of items (2-level factor, 1 vs. 3), and time-of-testing (2-level factor, morning vs. evening) and the between-subject factor group (2-level factor, young vs. old), as well as their interactions. A similar ART Anova was conducted on swap errors with the within-subject factors maintenance delay and time-of-testing and the between-subject factor group, as well as their interactions. Finally, the location error rate controlling for swap errors, or NIC (which can only be computed in 3-item conditions), was analysed with the same model as for the swap errors. Post hoc pairwise comparisons were conducted with contrasts adjusted using the Holm correction to account for multiple comparisons.

Concerning thermoregulation, we focused on the time course of skin temperature over the 24 h cycle. DPG analysis was performed using a mixed-design Anova with the between-subject factor group (2-level, young vs. old) and the within-subject factor time (24 time bins). Group and time were defined as fixed effects and subject as a random intercept. Body mass index (BMI) was added as a covariate as it was different between groups (see Table 1). Harmonic regressions were used to compare the phase and amplitude of the 24 h DPG modulation by group, based on the following model: Y **=** *β*0 + (*β*1 + *β*_Group1*(Group = 1)) * cos (2*pi*T/24) + (*β*2 + *β*_Group2*(Group = 1)) * sin (2*pi*T/24) + *β*3 (*t*), where Y is the dependent variable (DPG), *β*0 is the mean response, *β*1 and *β*2 are the coefficients of the cosine and sine harmonics, *β*_Group1 and *β*_Group2 are the coefficients of the categorical variable group, *β*3 is the coefficient of the linear trend, and T is the circadian period, set to 24 h. The categorical variable group is equal to 1 for old participants and 0 otherwise. From the model parameter estimates, we derived for each both amplitude (A= $\sqrt[{2}]{{\left(\beta 1+\;\beta \mathrm{\_Group}1*\left(\mathrm{Group}\;==1\right)\right)}^{2}+{\left(\beta 2+\beta \mathrm{\_Group}2*\left(\mathrm{Group}\;==1\right)\right)}^{2}\;}$) and phase (Phi = atan2 (ß2, ß1)/(2*pi)*360), where aran2 is the 2-argument arctangent function, for each group.

To investigate the hypothesis that vsWM performance is linked to the DPG level at the time of testing, a linear model assessed the impact of group (2-level, young vs. old) and DPG (continuous variable, as measured during computer testing, as mean DPG level between start and end of the task) on performance (4 models: identification, location, swap errors, NIC). Finally, difference scores were computed for DPG and performance metrics to assess the correlation between DPG and performance changes between sessions. To do so, we computed a differential DPG between testing times by subtracting the DPG during the morning test from the DPG during the evening test for each subject. The same calculation was applied to obtain the difference between evening and morning test performance for identification accuracy, absolute location error, and NIC. Kendall’s correlations were used to assess the correlation between the two difference scores. Finally, Kendall’s correlations were also applied to assess the link between DPG and cognitive performance (4 measures) during morning and evening sessions separately.

## Figures and Tables

**Table 1:** Demographic and questionnaire data by group (mean ± standard deviation). BMI: body mass index; PSQI: Pittsburg Sleep Quality Index [48]; MEQ: Morningness–Eveningness Questionnaire [49].

	Young (n = 18)	Old (n = 23)	Statistics
Age	23.9 ± 3.96	72.5 ± 6.14	*W* = 418, *p* < 0.001
Sex	13 women, 6 men	13 women, 9 men	*X*^2^ = 0.086, *p* = 0.769
BMI	21.3 ± 5.63	26.6 ± 3.19	*W* = 357, *p* < 0.001
Sleep quality (PSQI)	4.37 ± 1.64	3.96 ± 1.73	*W* = 184.5, *p* = 0.523
Chronotype (MEQ)	51.5 ± 9.32	63 ± 7.13	*W* = 348, *p* < 0.001
Wake-up time	07 h 53 ± 01 h 05	07 h 44 ± 01 h 01	*W* = 176, *p* = 0.817
Sleep onset time	23 h 47 ± 01 h 47	22 h 43 ± 00 h 26	*W* = 47.5, *p* < 0.001
Total sleep time	08 h 06 ± 01 h 27	09 h 01 ± 01 h 06	*W =* 258, *p* = 0.006

## Data Availability

The data underlying this article will be shared on request to the corresponding author.

## Supplementary Materials

The following supporting information can be downloaded at: https://www.mdpi.com/article/10.3390/clockssleep7020017/s1, Figure S1: Individual datapoints of the performance at the visuospatial object-binding task in old par-ticipants. (A,E) Identification accuracy; (B,F) Location errors (visual angle degree); (C) NIC, i.e., location error controlled for object-location swapping; (D) proportion of swap errors.
