# Supplementary material for: Uncorrelated Age-Related Changes in Visuo-Spatial Working Memory Binding and Thermoregulation
Source: Clocks Sleep. 2025 Mar 22;7(2):17. doi: 10.3390/clockssleep7020017 (PMC12192499; doi:10.3390/clockssleep7020017)
Supplement: Supplementary file 1 [file clockssleep-07-00017-s001.zip › clockssleep-3435753-supplementary.pdf]

clocks & sleep

MDPI

Supplementary Material

Uncorrelated age-related changes in visuo-spatial working memory binding and thermoregulation

Marine Dourte [1,2,3], Gregory Hammad [1], Christina Schmidt [1,2,*] and Philippe Peigneux [1,3]

1
Sleep and Chronobiology Laboratory, GIGA-CRC Human Imaging Unit, University of Liège, Liège, Belgium.
2
Psychology and Neuroscience of Cognition Research Unit (PsyNCog), Faculty of Psychology and Educational Sciences, University of Liège, Liège, Belgium.
3
UR2NF, Neuropsychology and Functional Neuroimaging Research Unit, affiliated at Center for Research in Cognition and Neurosciences and ULB Neurosciences Institute, Universite Libre de Bruxelles (ULB), Brussels, Belgium.
* Correspondence: christina.schmidt@uliege.be

Supplementary Material

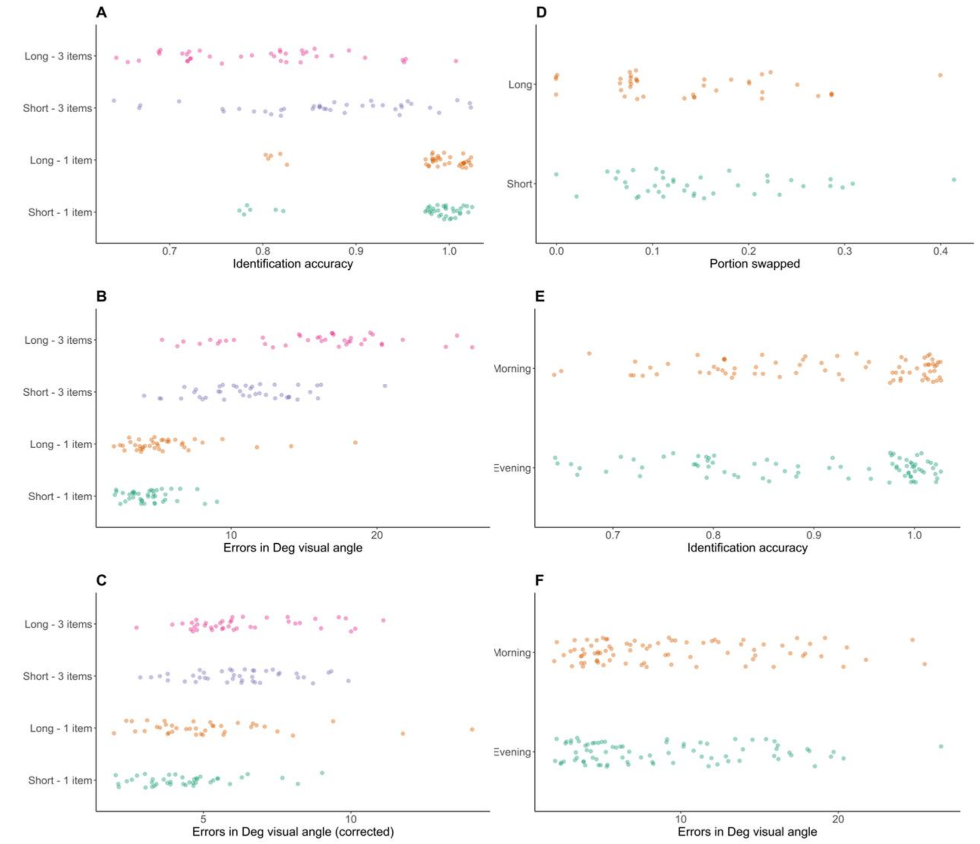

Figure S1. Individual datapoints of the performance at the visuospatial object-binding task in old participants. (A,E) Identification accuracy; (B,F) Location

 https://doi.org/10.3390/clockssleep7020017

errors (visual angle degree); (C) NIC, i.e., location error controlled for object-location swapping; (D) proportion of swap errors.

Disclaimer/Publisher's Note: The statements, opinions and data contained in all publications are solely those of the individual author(s) and contributor(s) and not of MDPI and/or the editor(s). MDPI and/or the editor(s) disclaim responsibility for any injury to people or property resulting from any ideas, methods, instructions or products referred to in the content.
